# Planar Body-Mounted Sensors for Electromagnetic Tracking

**DOI:** 10.3390/s21082822

**Published:** 2021-04-16

**Authors:** Marco Cavaliere, Herman Alexander Jaeger, Kilian O’Donoghue, Pádraig Cantillon-Murphy

**Affiliations:** 1 Tyndall National Institute, Dyke Parade, T12 R5CP Cork, Ireland; alexander.jaeger@tyndall.ie (H.A.J.); kilian.odonoghue@tyndall.ie (K.O.); p.cantillonmurphy@ucc.ie (P.C.-M.); 2 School of Engineering, University College Cork, College Road, T12 K8AF Cork, Ireland

**Keywords:** electromagnetic tracking, registration, image-guided surgery, inductive sensor, mutual inductance

## Abstract

Electromagnetic tracking is a safe, reliable, and cost-effective method to track medical instruments in image-guided surgical navigation. However, patient motion and magnetic field distortions heavily impact the accuracy of tracked position and orientation. The use of redundant magnetic sensors can help to map and mitigate for patient movements and magnetic field distortions within the tracking region. We propose a planar inductive sensor design, printed on PCB and embedded into medical patches. The main advantage is the high repeatability and the cost benefit of using mass PCB manufacturing processes. The article presents new operative formulas for electromagnetic tracking of planar coils on the centimetre scale. The full magnetic analytical model is based on the mutual inductance between coils which can be approximated as being composed by straight conductive filaments. The full model is used to perform accurate system simulations and to assess the accuracy of faster simplified magnetic models, which are necessary to achieve real-time tracking in medical applications.

## 1. Introduction

**Figure 1:**
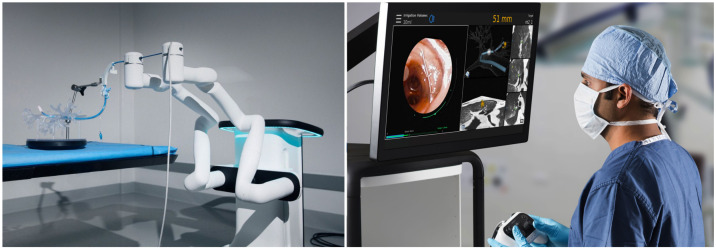
The Monarch robotic endoscopy platform (Auris Health Inc., Redwood City, CA, USA), as an example of image-guided surgery device.

Electromagnetic Tracking (EMT) is the gold standard technology for image-guided surgical interventions (Figure 1) without line of sight [1]. Existing applications include bronchoscopy [2], urology [3], orthopaedic surgery [4], catheter navigation [5,6].

**Figure 2:**
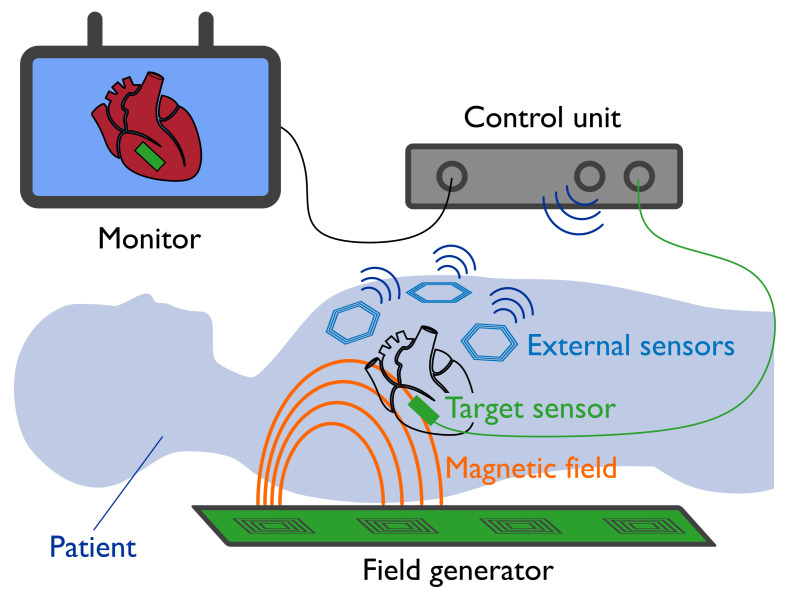
In electromagnetic tracking, a target sensor, attached to the instrument tip, measures the magnetic field generated by a set of transmitter coils. The signal is analysed by a control unit, which recovers sensor position and orientation from a known magnetic field model. The instrument is then visualised on an external monitor to assist the surgical operation. The use of external redundant sensors can help compensate for patient motion and magnetic field distortions.

The working principle behind EMT systems is detailed in Figure 2. Position and orientation information is based on the coupling between a field generator and a magnetic sensor. The magnetic model is the function that maps the position and orientation of the target sensor to the magnetic field measured at that location. The comparison between the model and the field measurement allows one to reverse the map and find the sensor pose in the working space. EMT system update-rate and latency directly depend on the model accuracy and calculation speed. Field generators are appositely shaped to generate a field that can be easily modelled (i.e., magnetic dipole [7,8,9], current sheets [10] or current filaments [11,12]). When an analytic model is not available, due to the shape of the transmitter coils or due to the presence of static field distortions, data-driven models are used which approximate the field values through Look-up Tables [13,14,15], Neural Networks [16,17,18] or multi-variable function interpolation [19,20,21].

This work focuses on the tracking of large inductive sensors on the centimetre scale. The field generator comprises a set of eight coils, as first presented in [12]. The transmitter coils are composed of straight filaments and embedded in a printed circuit board (PCB). Each coil is driven with a sinusoidal current at a different frequency. The pick-up coil measures a superposition the eight magnetic fields, and frequency-division multiplexing (FDM) is used to distinguish the eight signals. Based on this information, the five degrees of freedom (DoF) of the sensor coil are derived. Full system details can be found on the online project repository [22].

Planar coils integrated into patches and applied on the body of the patient, as shown in Figure 2, can improve the robustness of EMT for image-guided interventions. For example, a set of sensors placed around the operative region can track and compensate for respiratory or cardiac movements [23], or they can evaluate the field at multiple locations and perform the real-time compensation of field distortions by dynamically correcting the field model. Another possible application of the methods presented in this article involves the tracking of the planar transmitter coils of one or more field generators [24,25]. In this scenario, the reciprocal position between two boards can be determined by tracking the position of the coils of one board with respect to the other. This allows one to freely place two boards and enlarge the tracking region, with the ability to automatically determine the new position.

Inductive sensors measure the variation of the magnetic flux across the sensor area. While small-diameter sensors can be approximated with a single point without accuracy loss [26], larger-diameter sensors require the evaluation of the field at more points within the sensor area [27], in order to numerically integrate the magnetic flux measured by the sensor. The more accuracy that is required, the more points may be considered, resulting in a slower tracking algorithm.

A novel method is proposed which makes the flux calculation faster by the use of magnetic vector potential integration along the perimeter of the sensor coil.

The model is then compared to the single-point approximation, in terms of speed and accuracy. This work demonstrates the first EMT method based on the mutual coupling between straight-filaments, suited to large-area sensor coils.

## 2. Mutual Inductance between Large-Area Planar Coils

The mutual inductance between two coils is defined by the magnetic flux generated by one coil—which can be considered the transmitter coil Tx—that is linked to the loop of the second coil—or receiver coil Rx—per unit current $I_{Tx}$ (Equation (1)). In terms of magnetic vector potential, $A_{\mathrm{Tx}}$, the mutual inductance, $M_{TxRx}$, is also given by Equation (2), where $n_{\mathrm{Rx}}$ and $t_{\mathrm{Rx}}$ are the unit vectors perpendicular to the surface $d\Sigma_{Rx}$ and tangential to the loop $\Gamma_{Rx}$ respectively, and **r** is the position where $B_{\mathrm{Tx}}$ or $A_{\mathrm{Tx}}$ are evaluated:

(1) $$\begin{array}{cc} M_{TxRx} & =\frac{1}{I_{Tx}}\int_{\Sigma_{Rx}}B_{\mathrm{Tx}}\left(r\right)\;\cdot \;n_{\mathrm{Rx}}\;d\Sigma_{Rx} \end{array}$$

(2) $$\begin{array}{cc} \; & =\frac{1}{I_{Tx}}\int_{\Gamma_{Rx}}A_{\mathrm{Tx}}\left(r\right)\;\cdot \;t_{\mathrm{Rx}}\;d\Gamma_{Rx}. \end{array}$$

**Figure 3:**
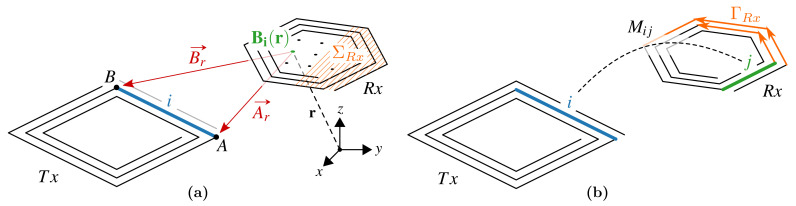
The mutual inductance between coil Tx and coil Rx can be (**a**) calculated from Equation (1) and numerically approximated by evaluation of the magnetic field generated by Tx across the area of Rx or (**b**) analytically calculated from Equation (2) as the sum of the partial mutual inductances between the straight filaments of the two coils.

The two approaches are illustrated in Figure 3.

Magnetic sensors used in EMT systems are usually of tiny dimensions, less than 1mm in diameter [28], which allows one to approximate the $\Sigma_{Rx}$ of Equation (1) by a single point.

For the tracking of larger-area inductive sensors, such as those studied in this work, the field may be evaluated at multiple points within the cross-sectional area of the sensor coil in order to perform a numerical integration, as depicted in Figure 3a. The number of evaluation points needed to achieve a given accuracy scales quadratically with the diameter of the receiving coil.

In this case, it becomes numerically more convenient to use Equation (2), where the integral is performed around the sensor loop and the number of segments that approximate the perimeter increases linearly with the diameter, as depicted in Figure 3b.

Expressing the Biot–Savart law in terms of magnetic vector potential [29,30], Equation (2) becomes:

(3) $$M_{TxRx}=\int_{\Gamma_{Rx}}\left[\frac{\mu_{0}}{4\pi }\int_{\Gamma_{Tx}}\frac{t_{\mathrm{Tx}}}{\left|r\right|}\;d\Gamma_{Tx}\right]\;\cdot \;t_{\mathrm{Rx}}\;d\Gamma_{Rx}=\frac{\mu_{0}}{4\pi }\int_{\Gamma_{Tx}}\int_{\Gamma_{Rx}}\frac{t_{\mathrm{Tx}}\;\cdot \;t_{\mathrm{Rx}}}{\left|r\right|}\;d\Gamma_{Rx}d\Gamma_{Tx},$$

which is referred as the *Neumann integral* [31,32].

If both Tx and Rx are composed of straight filaments, the double integral of Equation (3) can be split into the contributions given by all couples of two filaments [33]:

(4) $$M_{TxRx}=\sum_{i\in Tx}\sum_{j\in Rx}M_{ij},$$

where $M_{ij}$ is defined as the partial mutual inductance between filament *i* of coil Tx and filament *j* of coil Rx, as shown in Figure 3b, and will be analytically evaluated in Section 2.1.

### 2.1. Partial Mutual Inductance between Straight Wires at Any Angle

The Neumann integral (Equation (3)) performed between two straight filaments provides the partial mutual inductance between the filaments and can be solved analytically [34,35,36]. Analytical solutions presented in this article represent an improvement, in terms of accuracy and speed, over past works which used numerical integration [11,37].

**Figure 4:**
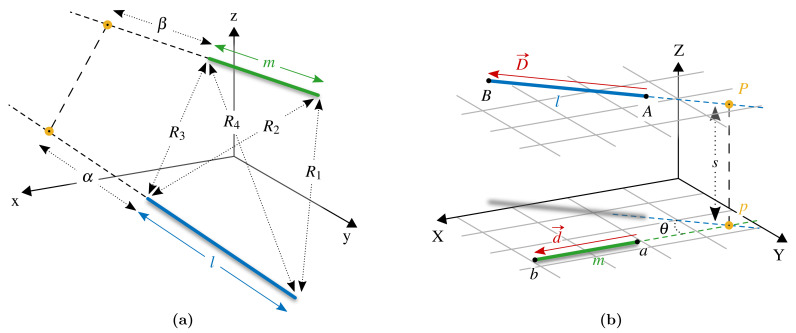
(**a**) Two straight filaments at any angle. For convenience, *l* is considered the transmitting filament which generates a magnetic field, *m* is the receiving filament, where a voltage is induced by the variable field. (**b**) A change in coordinates is performed in order to calculate the mutual inductance between the two wires.

In accordance to Figure 4, the following notation is defined. Points *A* and *B* are the three-dimensional vectors of the start and end points of the transmitting filament AB, respectively. It will be clear in Section 2.2 that *A* and *B* could be 3×n matrices of *n* transmitting vectors, such as the filaments composing a coil or a set of coils, allowing for fast vectorial implementation. Analogously, the receiving filament is defined by points *a* and *b*. The distances between the end-points of segments AB and ab are $R_{1}$, $R_{2}$, $R_{3}$, $R_{4}$, as indicated in Figure 4a.

Two direction vectors are found:

(5) $$\vec{D}=B-A;\;\vec{d}=b-a.$$

The lengths of the two filaments are $l=∥\vec{D}∥$ and $m=∥\vec{d}∥$.

For any two straight lines in the space, two planes exist which are parallel and contain these lines. The distance between the planes, *s*, corresponds to the minimum distance between the straight lines. The position of minimal distance is identified by point *P*. When projecting the filaments on the same parallel plane, the angle between the projections is θ.

The quantities α and β are the lengths of the prolongations of the filaments to the intersection point of minimal distance *P*, as indicated in Figure 4b.

Equation (6) gives the mutual inductance between two filamentary wires at any angle and not on the same plane [35,36]. The quantity Ω has the dimension of a solid angle and is given by Equation (7).

(6) $$\begin{array}{c} M_{ij}=\frac{\mu_{0}}{2\pi }\cos\left(\theta \right)\left[\left(\beta +m\right)\tanh^{-1}\left(\frac{l}{R_{1}+R_{4}}\right)-\right.\left.\beta \tanh^{-1}\left(\frac{l}{R_{2}+R_{3}}\right)+\right.\\ \left.+\left(\alpha +l\right)\tanh^{-1}\left(\frac{m}{R_{1}+R_{2}}\right)-\alpha \tanh^{-1}\left(\frac{m}{R_{4}+R_{3}}\right)-\frac{\Omega \;s}{2\sin(\theta )}\right], \end{array}$$

(7) $$\begin{array}{c} \Omega =\tan^{-1}\left(\frac{s^{2}\cos\theta +\left(\alpha +l\right)\left(\beta +m\right)\sin^{2}\theta }{sR_{1}\sin\theta }\right)-\tan^{-1}\left(\frac{s^{2}\cos\theta +\left(\alpha +l\right)\beta \sin^{2}\theta }{sR_{2}\sin\theta }\right)+\\ +\tan^{-1}\left(\frac{s^{2}\cos\theta +\alpha \beta \sin^{2}\theta }{sR_{3}\sin\theta }\right)-\tan^{-1}\left(\frac{s^{2}\cos\theta +\alpha \left(\beta +m\right)\sin^{2}\theta }{sR_{4}\sin\theta }\right). \end{array}$$

In order to generalise Equation (6), α and β may be defined with a positive sign if the prolongation towards point *P* is directed as the filament, negative otherwise [38].

### 2.2. Novel Implementation of the Formula

In this section, a new way to implement formula Equation (6) is described which considers the coordinates of the end-points of the filaments. The procedure is neat and it is well suited to vectorial input for the simultaneous calculation of the partial mutual inductances between one filament and many others.

As depicted in Figure 4, a change in coordinates is performed, to a reference system where the quantities required by Equations (6) and (7) can be easily computed. The new *X*-axis is defined to be aligned to the receiving filament. The unit direction vector is identified by $\vec{d}$, following normalisation:

(8) $$u_{X}=\vec{d}\;/\;∥\vec{d}∥=\vec{d}\;/\;m.$$

Axis *Z* is chosen to be perpendicular to both the filaments, i.e., perpendicular to the uniquely identified parallel planes containing the filaments, and axis *Y* is consequently found to complete the triplet:

(9) $$u_{Z}=\vec{D}\times \vec{d}\;/\;∥\vec{D}\times \vec{d}∥;\;u_{Y}=u_{Z}\times u_{X}.$$

It should be noted that $u_{Z}$ and $u_{Y}$ change for each transmitting filament and are matrices if multiple transmitting filaments AB are considered at the same time. Vectorisation is still possible for Equation (9) if the three vector components are computed by separate equations. The change in coordinates of the quantities of Figure 4 is performed by dot product with the new directions, e.g., for point $A=[A_{x}\;A_{y}\;A_{z}]$, the new component $A_{X}$ is found as:

(10) $$A_{X}=u_{X}x\;A_{x}+u_{X}y\;A_{y}+u_{X}z\;A_{z}.$$

In the new reference system XYZ, the distance between the straight lines is given by the Z-components:

(11) $$s=|A_{Z}-a_{Z}|.$$

The sine and cosine of the angle θ are:

(12) $$\sin\theta =\frac{D_{Y}}{l};\;\cos\theta =\frac{D_{X}}{l}.$$

Considering X and Y components only, the straight lines lay on the same plane and point *P* is their intersection:

(13) $$\begin{array}{cc} \; & P_{X}=\frac{D_{X}}{D_{Y}}\left(a_{Y}-A_{Y}\right)+A_{X},\\ \; & P_{Y}=a_{Y}. \end{array}$$

Finally, α and β, defined with a sign, as in [38], are:

(14) $$\begin{array}{c} \alpha =\frac{1}{l}\left[\left(A_{X}-P_{X}\right)\;D_{X}+\left(A_{Y}-P_{Y}\right)\;D_{Y}\right],\\ \beta =a_{X}-P_{X}. \end{array}$$

## 3. Sensor Manufacturing

In order to experimentally validate the formula proposed in this article, Section 2.2, a set of planar coils were manufactured on PCB. This section details the sensor coil design optimisation and the resulting sensor parameters.

### 3.1. Planar Sensor Coil Design

Two parameters are of primary importance for a coil used as an inductive sensor: the sensitivity (or sensor gain) and the Signal-to-Noise Ratio (SNR). Another important parameter is the frequency of resonance, related to the parasitic capacitance between the winding turns.

Sensor sensitivity is proportional to the total area enclosed by every turn of the coil. Sources of noise are thermal noise and the noise added by the signal amplifier. In particular, thermal noise is a function of the coil resistance, which is linear with the winding length.

**Figure 5:**
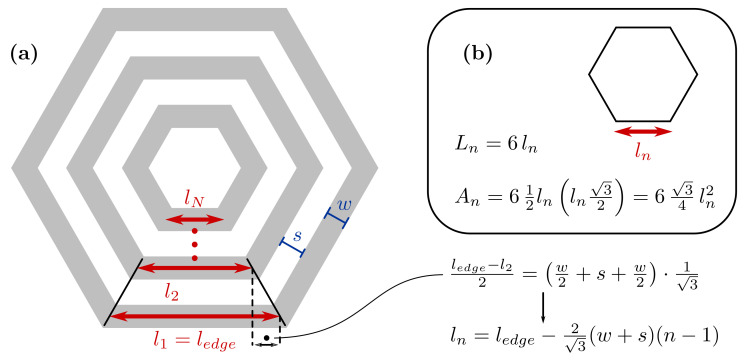
To calculate the cross-sectional area and the total length of a hexagonal planar coil, the spiral is approximated as a set of concentric hexagons. (**a**) The edge length, $l_{n}$, of the $n^{th}$ inner turn depends on the external edge, $l_{edge}$, and the trace width, *w*, and spacing, *s*. In the box (**b**), formulae for the equilateral hexagon perimeter $L_{n}$ and area $A_{n}$.

The coil total length and area are calculated as follows, for a hexagonal planar coil. Similar formulas apply for other planar shapes. The winding is approximated by concentric hexagons, as shown in Figure 5.

If $l_{edge}$ is the edge length of the external turn and *w* and *s* are the trace width and spacing, respectively, the edge length of the $n^{th}$ inner turn is computed as:

(15) $$l_{n}=l_{edge}-\frac{2}{\sqrt{3}}\left(w+s\right)\left(n-1\right),$$

where *n* starts from n=1 for the external turn.

Total winding length $L_{c}$ and area $A_{c}$ are:

(16) $$L_{c}=N_{layers}\;\cdot \;\sum_{n=1}^{N_{turns}}6\left[l_{edge}-\frac{2}{\sqrt{3}}\left(w+s\right)\left(n-1\right)\right]=N_{layers}\;\cdot \;6N_{turns}\left[l_{edge}-\frac{1}{\sqrt{3}}\left(N_{turns}-1\right)\left(w+s\right)\right],$$

and

(17) $$\begin{array}{ll} A_{c} & =N_{layers}\;\cdot \;\sum_{n=1}^{N_{turns}}6\frac{\sqrt{3}}{4}{\left[l_{edge}-\frac{2}{\sqrt{3}}\left(w+s\right)\left(n-1\right)\right]}^{2}\\ & =N_{layers}\;\cdot \;6\frac{\sqrt{3}}{4}N_{turns}\left[l_{edge}^{2}-\frac{2}{\sqrt{3}}\left(N_{turns}-1\right)l_{edge}\left(w+s\right)+\frac{2}{9}\left(N_{turns}-1\right)\left(2N_{turns}-1\right){\left(w+s\right)}^{2}\right], \end{array}$$

where $N_{turns}$ is the total number of turns and $N_{layers}$ is the number of PCB layers, assuming the same coil design is used for all the layers.

In Equations (16) and (17), the series sums are derived from the following identities:

(18) $$\sum_{n=1}^{N}n=\frac{N(N+1)}{2};\;\sum_{n=1}^{N}n^{2}=\frac{N(N+1)(2N+1)}{6}.$$

A copper coil is considered, with resistivity of $\rho_{Cu}=1.74\times 10^{-8}\;\Omega m$ and trace thickness t=34.79µm, corresponding to 1oz per square foot, as a standard PCB manufacturing specification. Coil ohmic resistance is:

(19) $$R_{c}=\rho_{Cu}\frac{L_{c}}{wt}.$$

Thermal noise RMS density is given by Equation (20):

(20) $$U_{nt}=\sqrt{4K_{B}TR_{c}}\;\left[\frac{\mathrm{nV}}{\sqrt{\mathrm{Hz}}}\right],$$

where $K_{B}=1.38\times 10^{-23}\;J\;/\;K$ is the Boltzmann constant and *T* is the ambient temperature, T=300K.

The inductive sensor signal requires amplification before sampling. A low-noise instrumentation amplifier INA163 (Texas Instruments, Dallas, TX, USA) is used in the system [26]. The INA163 introduces an input stage noise of $U_{nin}=1\;\mathrm{nV}\;/\;\sqrt{\mathrm{Hz}}$ and an output stage noise of $U_{nout}=60\;\mathrm{nV}\;/\;\sqrt{\mathrm{Hz}}$. In addition, an input noise current density of 0.8$\;\mathrm{pA}\;/\;\sqrt{\mathrm{Hz}}$ is declared, which was multiplied by the coil resistance, given by Equation (19), to get the noise density as a voltage. The amplifier was configured with a gain of $G_{amp}=500$.

Total noise magnitude depends on the signal bandwidth, Δf. The system uses FDM to demodulate the eight magnetic field measurements from the eight transmitter coils. The measured voltage was sampled at $F_{s}=100\;\mathrm{kHz}$, and 1000 samples were used to perform the frequency analysis [26]. Considering one of the eight frequencies, the target signal frequency is contained in a bin width of $\Delta f=F_{s}\;/\;1000=100\;\mathrm{Hz}$, which is the bandwidth of the noise added to the signal of interest.

Noise contributions sum quadratically, and the total noise is then computed as:

(21) $$U_{n}=\sqrt{\Delta f}\;\cdot \;\sqrt{{\left(G_{amp}\;\cdot \;U_{nt}\right)}^{2}+{\left(G_{amp}\;\cdot \;U_{nin}\right)}^{2}+{\left(G_{amp}\;\cdot \;R_{c}I_{nin}\right)}^{2}+U_{nout}^{2}}\;\;\left[V\right].$$

The voltage induced on the sensor is equal to the time derivative of the magnetic flux enclosed by the sensor coil. Assuming the magnetic field to be constant across the sensor area $A_{c}$, the magnitude of the signal measured is given by Equation (22), after amplification:

(22) $$U_{s}=G_{amp}\;\cdot \;2\pi fA_{c}B_{n}\;\;\left[V\right].$$

where *f* is the signal frequency and $B_{n}$ is the magnetic field normal component.

In the system configuration used in the following experiments, the eight sinusoidal fields have frequencies above f=20kHz, and a typical magnitude of the magnetic field normal component is above $B_{n}=0.1\;\mu T$. As the fields frequencies can be configured in a range between 20 and 50kHz [26], and the signal magnitude depends on the sensor position and orientation, the values considered for *f* and $B_{n}$ are just a reference used for the coil geometry optimisation and design process.

Finally, SNR is calculated from Equations (21) and (22):

(23) $$SNR=20\log_{10}\frac{U_{s}}{U_{n}}\;\;\left[\mathrm{dB}\right].$$

It can be seen that, in Equation (23), the coil area $A_{c}$ only appears in the numerator, and the winding length $L_{c}$ is only present in the denominator. As the ratio $A_{c}\;/\;L_{c}$ increases with the coil dimension, it follows that larger coils always provide larger SNR and signal $U_{s}$. Coil dimension is limited by the spatial constraints of the final application.

### 3.2. Coil Design Optimisation

In the optimisation process, the sensor coils external dimension, $D_{out}$, was fixed to 3 cm and SNR was optimised for the number of turns $N_{turns}$, trace width *w* and spacing *s*. The parameters *w* and *s* were constrained to a minimum value of t=0.127mm, corresponding to 5 mil, as it is a common PCB limit for standard production, while the maximum $N_{turns}$ was limited by what is physically manufacturable on a planar spiral of $D_{out}=3\;\mathrm{cm}$, for a given *w* and *s*.

The Matlab function fmincon was used to solve the constrained optimisation problem. For these constraints, the higher gain and SNR are obtained when trace width and spacing are minimised, w=s=0.127mm, and the number of turns is maximised.

It should be noted that this is not always the case, as for larger coil dimensions it would be more convenient to select a wider trace width, in order to reduce coil resistance and thermal noise, and to avoid filling all the space available, because inner turns could contribute more to noise than signal. In particular, when the signal is so large that the amplifier noise can be neglected, thermal noise would dominate in the coil design for SNR optimisation.

**Figure 6:**
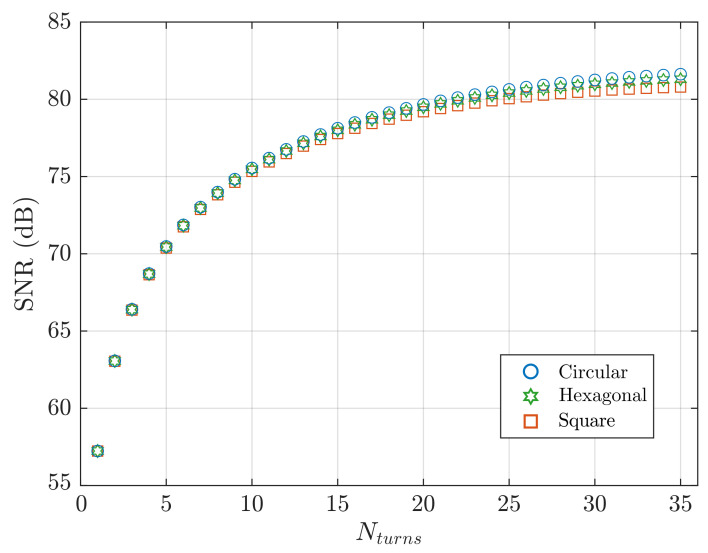
SNR predicted from Equation (23). Circular, hexagonal and square planar coil shapes are compared, with variation in turn count. The three planar coils have the same footprint area (5.85 ${\mathrm{cm}}^{2}$) of a hexagon with a diagonal of 3 cm.

Three coil shapes were compared: a hexagonal coil with a diagonal of 3cm, and a circular and square planar coils with the diameter and the edge, respectively, defined to give the same footprint area as the hexagonal coil. SNR calculated from Equation (23) is plotted in Figure 6, with variation in turn count.

The circular shape provides higher SNR for two reasons: (1) when more turns are added, the cross-sectional area increases more for a circular winding than for a hexagonal or square winding, yielding a higher signal magnitude; (2) a circular coil has the maximum enclosed area for a given winding length, minimising the resistance and the thermal noise.

**Figure 7:**
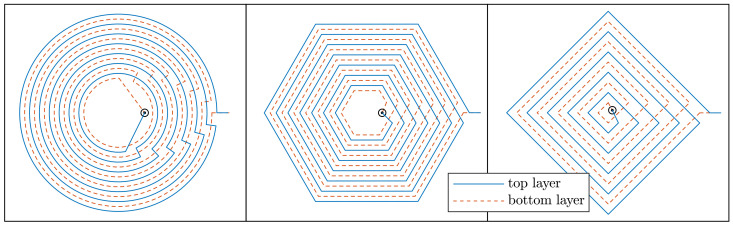
Optimisation provided the same value for trace width and spacing, for PCB planar coils on the centimetres scale. To minimise the parasitic capacitance of the winding, it is convenient to shift the footprints of subsequent layers, so that the traces do not overlap.

The parasitic capacitance of planar coils is mainly due to the electrical coupling between overlapping traces of different layers [39,40]. To decrease the parasitic capacitance of the coil and maximise the self resonance frequency, the footprints of subsequent layers were designed with shifted traces, as illustrated in Figure 7.

### 3.3. Coil Measured Parameters

**Figure 8:**
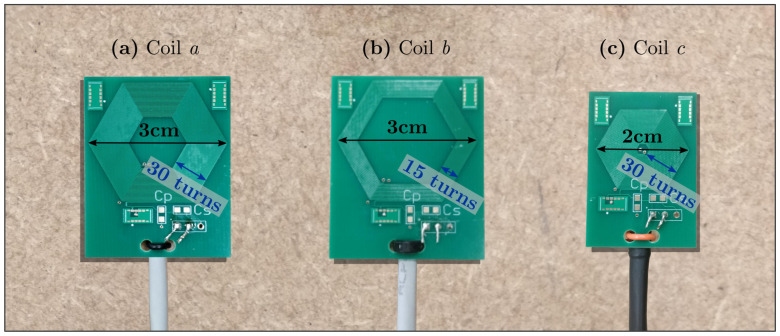
Three hexagonal sensors were manufactured on a 4-layer PCB. (**a**) The reference coil *a* has a diagonal, $D_{out}$, of 3 cm and 30 turns per layer. (**b**) Another version with 15 turns, and (**c**) a smaller coil with $D_{out}=2\;\mathrm{cm}$, were manufactured to validate the formulas used in the coil design procedure.

In order to experimentally validate Equation (6), which applies to coils composed of straight filaments, the hexagonal planar coil of Figure 8a was PCB manufactured. A hexagonal shape provides higher SNR than a square coil (Figure 6) and can be entirely modelled by six filaments per turn. Coil parameters are: w=s=0.127mm, $N_{turns}=30$, $D_{out}=3\;\mathrm{cm}$, $N_{layers}=4$. Two other coils were used for comparison, the first (Figure 8b) having less turns, $N_{turns}=15$, and the second (Figure 8c) with a smaller diagonal dimension, $D_{out}=2\;\mathrm{cm}$.

Coil resistance was calculated with Equation (19). The self inductance can by estimated by Equation (4), if the transmitting and receiving filaments are taken from the same coil. For the three coils of Figure 8, predicted ohmic resistance and self inductance are:

(24) $$\begin{array}{cc} R_{a} & =30.47\;\Omega ;\;R_{b}=18.36\;\Omega ;\;R_{c}=16.30\;\Omega .\\ L_{a} & =304.2\;µH;\;L_{b}=133.3\;µH;\;L_{c}=111.2\;µH \end{array}$$

Coil resistance and self inductance were measured at 1kHz. Experimental values for coils *a*, *b* and *c* of Figure 8 are:

(25) $$\begin{array}{cc} R_{a} & =37.0\;\Omega ;\;R_{b}=22.8\;\Omega ;\;R_{c}=18.6\;\Omega ;\\ L_{a} & =289.4\;µH;\;L_{b}=121.2\;µH;\;L_{c}=98.2\;µH, \end{array}$$

to be compared with Equation (24).

Inductive sensor theoretical sensitivity to a sinusoidal magnetic field is:

(26) $$k_{s}=2\pi A_{c}\;\left[\frac{V}{T\mathrm{Hz}}\right]$$

where the coil area is calculated as per Equation (17). For a reference, commercial sensors typical sensitivity is on the order of 0.1V/THz [41]. The sensitivity of the three sensors under analysis was not directly measured, but the variation in the output sensor voltage was measured for coil *b* and *c* relative to coil *a*. Results are summarised in Table 1.

## 4. Mutual Inductance Measurement

Coil *a* of Table 1, shown in Figure 8a, was selected as preferred sensor model for the other experiments presented in the next sections of this article.

**Figure 9:**
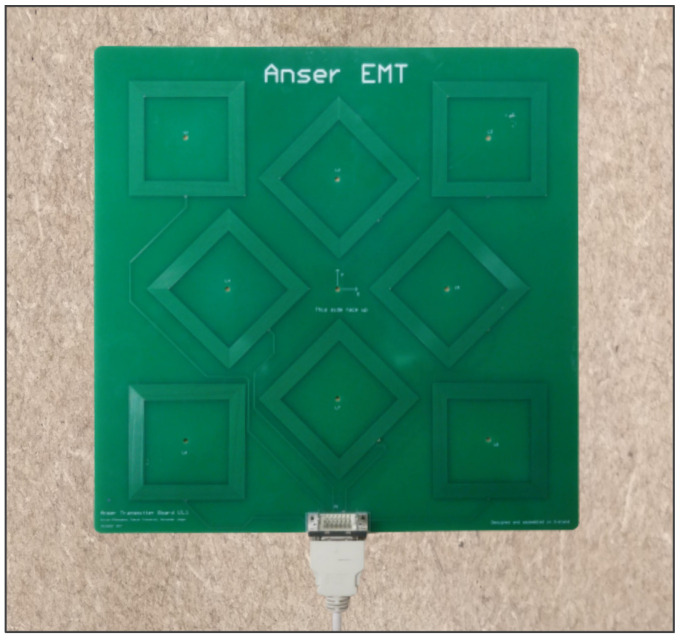
The field generator comprises a set of eight planar transmitter coils, as first presented in [12]. Each coil is driven with a sinusoidal current at a different frequency and the sensor coil uses frequency-division multiplexing (FDM) to distinguish the eight signals.

A set of mutual inductances were measured between coil *a* and the eight transmitter coils of the field generator, shown in Figure 9.

**Figure 10:**
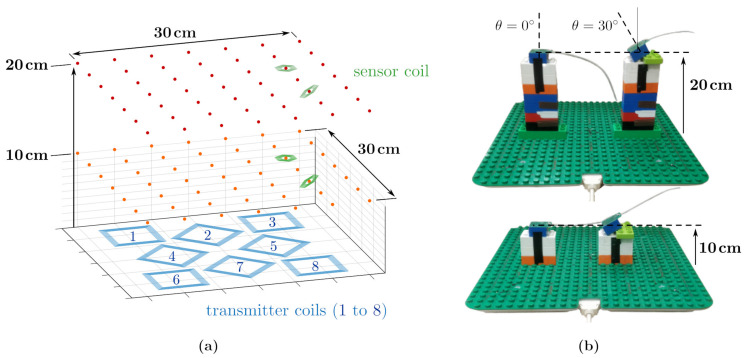
(**a,b**) The mutual inductance was measured on a grid of 7×7 points at 10 cm and 20 cm from the planar field generator, for two sensor orientations: pitch angle $\theta =0^{\circ }$ and $\theta =30^{\circ }$. At every test point, the eight mutual inductances between the sensor coil and the eight transmitter coils are considered, leading to a total of 49×4×8=1568 mutual inductances experimentally evaluated.

The receiving coil was positioned on a grid of 7×7 test points laying on a horizontal plane. The experiment was repeated for two distances from the planar transmitter board, 10cm and 20cm, and for two sensor orientations, vertically oriented and with an angle of $30^{\circ }$ from the vertical axis, as shown in Figure 10a.

Duplo blocks (The Lego Company, Billund, Denmark) were used to precisely locate the sensor in known positions (Figure 10b). At every test position, 100 measurements were collected and the average was considered, in order to reduce random error.

For each sensor position and orientation, the eight mutual inductances between sensor and the transmitter coils of the field generator were measured, leading to a total of 1568 mutual inductance values experimentally collected, for the 98 different positions and two sensor orientations.

Mutual inductances at test-points were also simulated with the analytical model proposed in Section 2. PCB traces of the transmitting and the receiving planar coils were approximated as 0-radius straight filaments. Previous works demonstrated that this assumption is true almost everywhere apart from the close proximity (approximately less than five times the trace width) to the conductor [42,43].

The model takes into account all the filaments composing the square transmitter coil and all the filaments of the hexagonal sensor coil, to compute the mutual inductance between the two coils as the sum of the contributions given by every pair of filaments.

**Figure 11:**
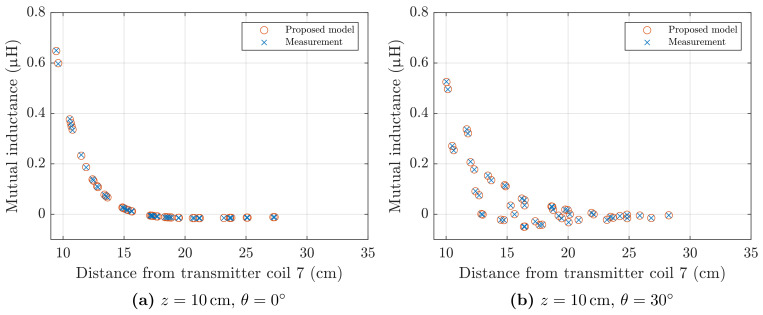
Mutual inductance between coil *a* of Figure 8a, and transmitter coil 7 of the field generator, as defined in Figure 10a. Experimental values are compared to those predicted by the analytical model of Section 2. (**a**) Test points at 10 cm from the planar field generator, pitch angle $\theta =0^{\circ }$. (**b**) Test points at 10 cm from the planar field generator, pitch angle $\theta =30^{\circ }$.

Figure 11 shows the mutual inductance between the sensor coil *a* and transmitter coil 7, as defined in Figure 10a, for the grid of points at z=10cm and the two sensor orientations. The values experimentally measured are compared to the values predicted by the formula proposed in this article, Section 2.

Results of the four tests performed are summarised in Table 2. Each of the four cases includes 49×8=392 inductance values. The average measured mutual inductance, $M_{avg}$, the root-mean-square error (RMSE) and the maximum error (MAXE) between the measurement and the model are reported.

## 5. System Simulation

Mutual inductance calculation provides the mutual inductance between transmitter and receiver coils, based on the relative position between the two coils.

The positioning algorithm aims to solve the inverse problem: finding the sensor position and orientation, given the set of mutual inductances measured between the eight transmitter coils of the field generator, Figure 9, and the sensor coil, Figure 8a.

The problem was solved in a least-squares sense, with cost function:

(27) $$\hat{x}=\underset{x}{\arg\min}\sum_{i=1}^{8}{\left(V_{meas,i}-V_{model,i}\left(x\right)\right)}^{2}$$

where $\hat{x}={\left[\hat{x}\;\hat{y}\;\hat{z}\right]}^{T}$ is the estimated sensor position, $V_{meas,i}$ is the voltage measurement, related to transmitter coil *i*, and $V_{model,i}$ is the signal predicted by the magnetic model.

### 5.1. Magnetic Model

The transmitter board comprises eight square planar coils of 25 turns, for a total of 8×4×25=800 filaments, as shown in Figure 9. The receiving sensor is a hexagonal planar coil of 30 turns, composed by 6×30=180 filaments, as shown in Figure 8a. The *full* magnetic model accounts for the magnetic mutual coupling between all the pairs of transmitting and receiving filaments, which is given by Equation (6). Vectorisation is of massive importance for speed, because a total of 800×180= 144,000 filament couples has to be considered.

The optimisation algorithm was implemented on Matlab, (Mathworks, Natick, MA, USA), running on an HP EliteBook 840 G3 (HP Inc., Palo Alto, CA, USA) with a Core i7-6500U 2.5 GHz processor and 8Gb of RAM. Matlab function *lsqnonlin* was used to solve Equation (27), resulting on an average solution time on the order of seconds. Therefore, the *full* model is not suitable to real-time tracking applications, which require update-rates at least on the order of tens of Hz [44].

Two simplified but faster models are proposed and analysed. The first model considers only one turn for the receiving coil, or *six filaments*. A further simplification does not take into account the receiving coil area, and evaluates the magnetic field at a *single point* at the middle. In both the cases, the transmitter coils are not simplified and all the 800 filaments are considered.

### 5.2. Dependence on Distance

The Accuracy of the simplified *6-filament* and *single-point* models was tested for the mutual inductance calculation between two coils, with variation in the distance, using the *full model* as a ground-truth reference.

**Figure 12:**
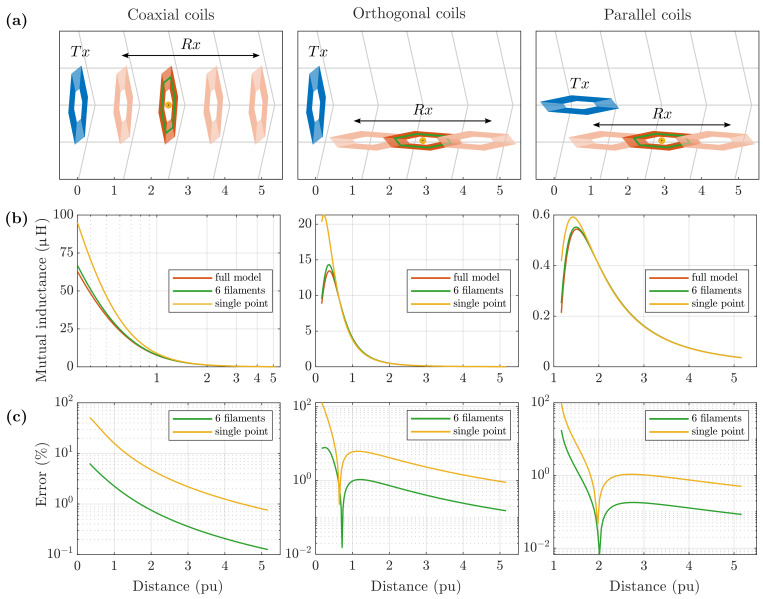
(**a**) A transmitter coil, Tx, and a receiver coil, Rx, at increasing distance, for three orientation configurations. Plots are on a *per unit* (p.u.) scale, referring to the coil diameter. (**b**) The mutual inductance was calculated with the *full* model, *6-filament* and *single-point* approximations. (**c**) The relative error falls below 1% at a distance of larger than 2 p.u. and 5 p.u. for the *6-filament* and *single-point* models, respectively, for all the three orientations considered.

Two hexagonal planar coils of 3 cm diagonal were considered. Mutual inductance was calculated at increasing distance, for different reciprocal orientations, as drawn in Figure 12a. The yellow dot indicates the position of the point of evaluation for the *single-point* model and the green hexagon highlights the single turn considered in the *6-filament* model.

Mutual inductances calculated with the *full*, *6-filament* and *single-point* models are plotted in Figure 12b, on a per-unit axis, relative to the hexagonal coil diagonal. Figure 12c shows the relative percentage error of the two simplified models against the exact model reference.

As expected, relative error decreases with distance, as model approximations become less important. The error depends on coil orientation, but some general observations are true for all three orientation cases considered.

For the *single-point* model, the relative error becomes smaller than 1% at a distance of approximately five times the coil diagonal. For a coil of 3 cm, such as coil *a* under analysis, it corresponds to 15 cm.

For the *6-filament* model, the relative error maintains almost constantly five times smaller than the *single-point* error, becoming less than 1% below a distance of 2 p.u.

### 5.3. Static Tracking Simulation

Virtual tracking simulations can be used to validate magnetic models for EMT [45]. The sensor is positioned at a number of locations within the tracking region, where the magnetic field is given by an accurate field simulation. The magnetic model being tested is used to infer the sensor position, starting from the simulated magnetic measurement.

With reference to Equation (27), the voltage measurement $V_{meas,i}$ was simulated using the *full* model described in Section 5.1, while $V_{model,i}$ is the signal predicted by the magnetic model being tested.

To compare the *6-filament* and the *single-point* models, two virtual static tracking accuracy tests were performed. In the first test, position errors due to model approximations were studied. The magnetic sensor was virtually placed at 512 random positions between 1 and 11 cm from the transmitter board.

In the second test, random white noise was added to the simulated signal, to analyse the robustness of the two models. The sensor was placed at 512 random locations in a cube of 30×30×30cm. The simulated noise magnitude was considered fixed, not dependent on the signal, representing an external noise source, meaning that SNR is not constant and depends on the signal magnitude. Test points further from the field generator have a smaller signal and, thus, present a smaller SNR.

The test was repeated using two different Gaussian noise magnitudes, in order to evaluate the influence of noise on tracking error. For the two cases, the SNR values computed as an average on all the test points are 65 dB and 50 dB respectively.

### 5.4. Static Tracking Simulation Results

The position error is computed as the Euclidean distance between the test position $x={\left[x\;y\;z\right]}^{T}$, used to simulate the magnetic measurement, and the position solved by the tracking algorithm, $\hat{x}={\left[\hat{x}\;\hat{y}\;\hat{z}\right]}^{T}$:

(28) $$E=\sqrt{{\left(x-\hat{x}\right)}^{2}+{\left(y-\hat{y}\right)}^{2}+{\left(z-\hat{z}\right)}^{2}}.$$

**Figure 13:**
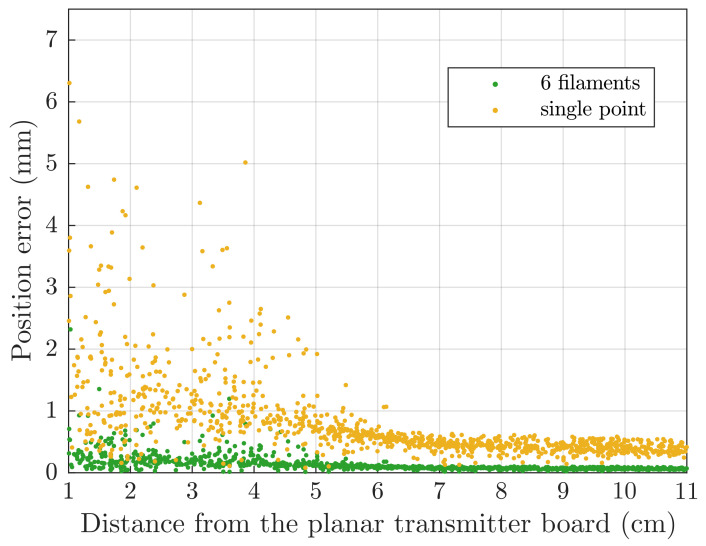
Static accuracy test simulation with no added noise. Position errors are due to model approximations. The two simplified models presented in Section 5.1 are compared.

Scatter plot of the error is shown in Figure 13, where it can be seen that the *single-point* approximation is not accurate in the proximity of the field generator. However, it can be used for large-area coils, such as the one under analysis, at distances above 6 cm, with sub-millimetre errors.

For more accurate tracking near the field generator, the *6-filament* model can be used, but as reported in Table 3, it takes more time for computation on average.

Computation time is calculated as the average time for the solution of each of the 512 test points. Average solution time, root-mean-square error (RMSE) and mean error (ME) are detailed in Table 3, for the two models described.

**Figure 14:**
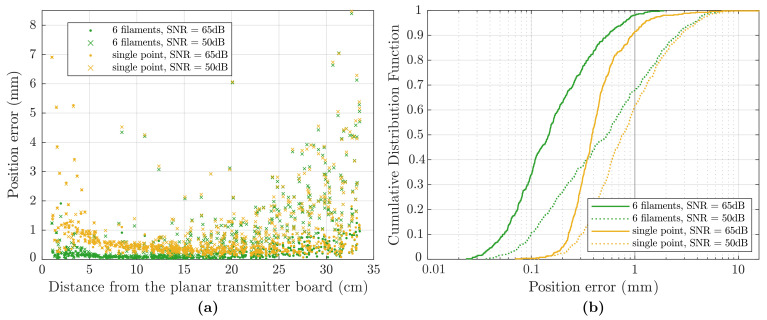
(**a**) Static accuracy test simulation with noisy data, two noise levels are compared. Position errors depend on model approximations and on noise magnitude. (**b**) Cumulative distribution function of the position error, for the two magnetic models considered and average noise levels of 50 and 65 dB.

For the results of Figure 14, Gaussian noise was added to the simulated signal, $V_{meas}$ of Equation (27). Compared to the no-noise simulation of Figure 13, average tracking error increases, as the noisy measured signal deviates from the magnetic model.

Error is plotted in Figure 14a. Test points at higher distance have a lower signal and SNR, resulting in higher position error. It can be noticed that, for the compared *6-filament* and the *single-point* models, solutions are similar for positions far from the transmitter board, where SNR is low and the error is mainly due to the externally added noise. On the other hand, for positions near the transmitter board different noise levels have less influence, while the source of error is due to the model approximations.

The cumulative distribution function of the error is plotted in Figure 14b and shows that, in the lower noise case, with an average SNR of 65 dB, almost all the points obtained with the *6-filament* model and approximately 90 % of the points for the *single-point* model exhibit sub-millimetre errors. If the external noise is increased, with an average SNR of 50 dB, the percentages of the points with sub-millimetre errors decrease to approximately 70 % and 60 % respectively.

## 6. Experimental Tracking Accuracy Test

A real electromagnetic tracking test was performed to estimate the static positional accuracy of planar coil *a*, using the magnetic model proposed in this article.

The four test cases described in Section 4 and visualised in Figure 10 were repeated, where in this case the real field measurements, $V_{meas}$, were used to estimate the position of the sensor, from Equation (27). The *6-filament* and the *single-point* magnetic models, previously presented, are compared in terms of tracking accuracy and speed.

**Figure 15:**
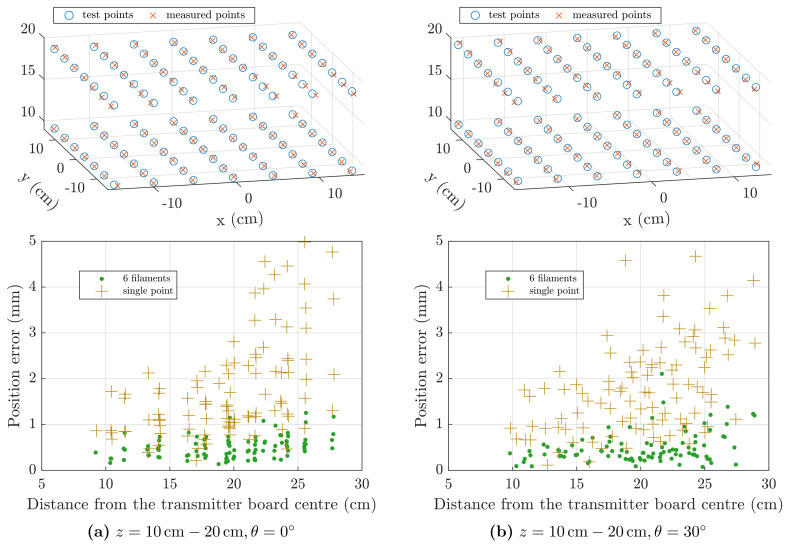
Static accuracy test with real data. In the first row, test points and positions solved using the *single-point* model. In the second row, scatter plot of the position error, for the *6-filament* and *single-point* models. Columns (**a,b**) show the results for different sensor orientations, $\theta =0^{\circ }$ and $\theta =30^{\circ }$, respectively.

Electromagnetic tracking test results are presented separately in Figure 15 for the two sensor orientation cases, $\theta =0^{\circ }$ and $\theta =30^{\circ }$. The first row of Figure 15 shows the two grids of 49 points at z=10cm and z=20cm, together with the positions estimated by the *single-point* model.

The second row of Figure 15 shows the scatter plot of the total position error, computed as for Equation (28), against the distance from the centre of the field generator. It can be seen that the *6-filament* model yielded better accuracy than the *single-point* model, with errors below 1mm for almost all the test points. Errors of the *single-point* model are below 5mm. Positions further from the centre-point show, on average, larger errors, due to signal attenuation and decreased SNR.

Average solution time, root-mean-square error (RMSE) and mean error (ME) are detailed in Table 4, for the four test cases and the two magnetic models described.

## 7. Discussion

A method for the calculation of the mutual inductance between coils composed of straight filaments was presented, and a new formulation for the partial mutual inductance between arbitrarily oriented straight filaments was proposed.

Coil design demonstrated that planar coils on the centimetre scale are optimised when the minimum track width and spacing are chosen, and the number of turns is maximised. The circular shape is optimal for SNR optimisation, Figure 6, but a hexagonal coil shape was selected, because it can be entirely modelled by straight filaments. In Table 1, measured coil resistance, inductance and sensitivity variation are compared with the values predicted in the design stage.

A hexagonal planar coil with a diameter of 3 cm was PCB manufactured. A set of mutual inductances was measured between the sensor coil and the eight transmitter coils of the field generator. Experimental values validated the model proposed in Section 2. A selection of the results is plotted in Figure 11, and the average error is reported in Table 2.

The tracking algorithm was implemented in Matlab, (Mathworks, Natick, MA, USA), with an average solution time in the order of seconds, when all the pairs of transmitting and receiving filaments were considered. To allow for real-time EMT, two simplified models were introduced, where the sensor coil was approximated by *six filaments* or by a *single point* respectively.

Virtual tracking error is plotted in Figure 13, where it can be seen that the *single-point* model provides sub-millimetre positioning errors at distances larger than 6 cm from the transmitter board, while the *6-filament* model reaches the same level of accuracy even for closer positions.

Four EMT static tests were performed, varying the sensor orientation and distance from the field generator. As reported in Table 4, using the *6-filament* model the position error is three to four times smaller than using the *single-point* model, but the average solution time is approximately 0.15 s, compared to 0.01 s for the *single-point* model.

## 8. Conclusions

Magnetic sensors are commonly used for electromagnetic tracking in image-guided interventions. The use of redundant sensors can help to detect and minimise the effect of magnetic distortions. One additional application is the surface tracking of breathing motion to compensate for registration errors.

Large-area planar sensors show high sensitivity, even in the absence of a magnetic core. They can be printed on flexible PCB, and embedded in adhesive patches, with reduced costs and high precision manufacturing and repeatability.

This paper analyses the tracking performances of planar coils on the centimetre scale. An accurate magnetic model is proposed and experimentally validated. While the model was too slow for effective real-time EMT, it proved useful to perform sensor design and calibration and to run tracking simulations.

The introduction of model simplifications increased the calculation speed, and the real-time tracking of large-area planar sensors was demonstrated with sub-millimetre positioning errors.

Future work will investigate the use of PCB printed planar sensors to record the registration volume deformation and compensate the target sensor position for patient movements. In another study, the sensors will collect real-time measurements of the magnetic field at known positions, in order to recognise and compensate for metallic distortions.

## Figures and Tables

**Table 1:** Coil resistance and self inductance predicted and measured at 1kHz, for the three coils of Figure 8. Sensitivity predicted by Equation (26) and signal variation experimentally measured between the three coils.

	Coil *a*	Coil *b*	Coil *c*
Coil dimension	$D_{out}=3\;\mathrm{cm}$,	$D_{out}=3\;\mathrm{cm}$,	$D_{out}=2\;\mathrm{cm}$,
	30 turns	15 turns	30 turns
Resistance (Equation (24)) $\;\left[\Omega \right]$	30.47	18.36	16.3
Resistance meas. $\;\left[\Omega \right]$	37	22.8	18.6
Inductance (Equation (6)) $\;\left[µH\right]$	304.2	133.3	111.2
Inductance meas. $\;\left[µH\right]$	289.4	121.2	98.2
Sensitivity (Equation (26)) $\;\left[\frac{V}{T\mathrm{Hz}}\right]$	0.239	0.166 (69.4%)	0.077 (32.4%)
Sensitivity meas. $\;\left[\%\right]$	—	66.4%	30.3%

**Table 2:** Each test includes 392 mutual inductance values, with average $M_{avg}$. RMSE and MAXE are calculated between the measurement and the model proposed in Section 2.

Test	$M_{\mathrm{avg}}$ [nH]	RMSE [nH]	MAXE [nH]
z = 10 cm, theta = $0^{\circ }$	85.808	1.354	6.585
z = 10 cm, theta = $30^{\circ }$	78.061	0.979	4.343
z = 20 cm, theta = $0^{\circ }$	30.428	0.174	0.549
z = 20 cm, theta = $30^{\circ }$	26.990	0.144	0.490

**Table 3:** Speed and accuracy of the *6-filament* and the *single-point* models used in a simulated electromagnetic tracking experiment. Scatter plot of the error is shown in Figure 13. Computation time is calculated as the average solution time for 512 random test points.

Model	Time [s]	Distance from Board [cm]	RMSE [mm]	ME [mm]
6-filament	0.052	1–6	0.2615	0.1903
		6–11	0.0754	0.0733
single-point	0.007	1–6	1.4083	1.1024
		6–11	0.4485	0.4357

**Table 4:** Speed and accuracy of the *6-filament* and the *single-point* models used in a real electromagnetic tracking experiment. Scatter plot of the error is shown in Figure 15. Computation time is calculated as the average solution time for the 49 test points.

Test	Model	Time [s]	RMSE [mm]	ME [mm]
$z=10\;\mathrm{cm},\theta =0^{\circ }$	6-filament	0.1420	0.54	0.49
	single-point	0.0091	1.54	1.31
$z=10\;\mathrm{cm},\theta =30^{\circ }$	6-filament	0.1483	0.63	0.52
	single-point	0.0094	1.92	1.57
$z=20\;\mathrm{cm},\theta =0^{\circ }$	6-filament	0.1541	0.58	0.52
	single-point	0.0098	2.47	2.23
$z=20\;\mathrm{cm},\theta =30^{\circ }$	6-filament	0.1488	0.57	0.48
	single-point	0.0097	2.29	2.07

## Data Availability

Not applicable.

## Abbreviations

The following abbreviations are used in this manuscript:

EMT	ElectroMagnetic Tracking
PCB	Printed Circuit Board
FDM	Frequency-Division Multiplexing
SNR	Signal-to-Noise Ratio
RMSE	Root-Mean-Square Error
ME	Mean Error
